# Test Statistics for the Identification of Assembly Neurons in Parallel Spike Trains

**DOI:** 10.1155/2015/427829

**Published:** 2015-03-08

**Authors:** David Picado Muiño, Christian Borgelt

**Affiliations:** European Centre for Soft Computing, Edificio Científico Tecnológico, Gonzalo Gutiérrez Quirós, s/n, 33600 Mieres, Spain

## Abstract

In recent years numerous improvements have been made in multiple-electrode recordings (i.e., parallel spike-train recordings) and spike sorting to the extent that nowadays it is possible to monitor the activity of up to hundreds of neurons simultaneously. Due to these improvements it is now potentially possible to identify assembly activity (roughly understood as *significant* synchronous spiking of a group of neurons) from these recordings, which—if it can be demonstrated reliably—would significantly improve our understanding of neural activity and neural coding. However, several methodological problems remain when trying to do so and, among them, a principal one is the combinatorial explosion that one faces when considering all potential neuronal assemblies, since in principle every subset of the recorded neurons constitutes a candidate set for an assembly. We present several statistical tests to identify assembly neurons (i.e., neurons that participate in a neuronal assembly) from parallel spike trains with the aim of reducing the set of neurons to a relevant subset of them and this way ease the task of identifying neuronal assemblies in further analyses. These tests are an improvement of those introduced in the work by Berger et al. (2010) based on additional features like spike weight or pairwise overlap and on alternative ways to identify spike coincidences (e.g., by avoiding time binning, which tends to lose information).

## 1. Introduction

The principles of neural coding and information processing in biological neural networks are still not well understood and are the topic of ongoing debate. As a model of network processing, neuronal assemblies were proposed in [1], which are intuitively understood as groups of neurons that tend to exhibit synchronous spiking.

In recent years considerable improvements have been made in multiple-electrode recordings and spike sorting (see, e.g., [2, 3]) that allow monitoring the activity of up to hundreds of neurons simultaneously. These improvements open the possibility of identifying neuronal assemblies from multiple-electrode recordings using statistical data analysis techniques. However, several methodological problems remain when trying to do so and, among them, a principal one is the combinatorial explosion that we face when considering all potential neuronal assemblies (since in principle every subset of the recorded neurons constitutes a candidate set for an assembly). For this reason, most studies that deal with temporal spike correlation still resort to analyzing only pairwise interactions (see, e.g., [4–7]), thus considerably reducing the computational complexity of such task. There are approaches in the literature that try to infer higher-order correlation and potential assembly activity by building primarily on these pairwise interactions (see, e.g., [8–11]) but, although they can sometimes provide a hint of higher-order correlation and even closely identify assembly activity (provided it is sufficiently pronounced), higher-order correlations need to be checked directly in order to properly identify neuronal assemblies, mostly for two reasons: first, to make sure that the activity reported is actually that of an assembly and not just of several overlapping pairs and, second, to increase the sensitivity for assembly activity as pairwise tests may not be affected sufficiently by assembly activity (see, e.g., [12, 13]). Some approaches already do so (see, e.g., [14–16]) yet they are all generally limited to a small number of neurons. Others presented in some of our recent companion papers (see, e.g., [17–19]) push this limitation by employing frequent item set mining methodology and algorithms to ease and speed up the search through all the candidate sets for potential assemblies, yet combinatorial explosion remains a fundamental problem (especially since statistical tests aiming at identifying assembly activity often rely on randomization or surrogate data approaches, which drive up the computational complexity even further).

In this paper we present several statistical tests to identify individual assembly neurons (i.e., neurons that are part of an assembly). Our tests extend and considerably improve those presented in [20], which were based on time binning and were mostly intended to identify* exact* (or almost exact) spike synchrony—which is more a theoretical simplification for modelling purposes rather than a realistic assumption. With the new tests introduced in this paper we can do much better: first, we introduce new features into the tests that make them more sensitive (like, e.g., spike weights or pairwise overlap of spikes) and, second, we introduce new ways to identify spike coincidences (i.e., we introduce alternatives to time binning to avoid the loss of detectable synchronous activity). The main motivation of our tests is to reduce the set of neurons only to a relevant subset of them and in this way ease the task of identifying neuronal assemblies in further analyses (i.e., by reducing the total number of neurons to those that tested positive in our approach, the combinatorial explosion can be reduced significantly). The idea of all tests that we present in this paper is fairly simple: we evaluate whether an individual neuron is involved* significantly* more often in some correlated-spiking event (that depends on the particular test) than it would be expected by chance under the assumption of noncorrelation (i.e., independence). In order to assess significance we estimate the distribution of our test statistics by means of randomized trials (i.e., collections of parallel spike trains): modifications of our original data that are intended to keep all its essential features except synchrony for the neuron we are testing.

The paper is structured as follows: in Section 2 we mainly introduce some notation that we will be using throughout the paper and briefly discuss the notion of* spike synchrony*, central to our research. In Section 3 we introduce our test statistics to identify assembly neurons. First, in Section 3.1 we provide four statistical tests that rely on a window-based approach to identify spike coincidences. Technically speaking, different collections of windows provide different ways of counting spike coincidences and thus different tests. We consider in our evaluations two collections of windows: the first one we consider is a partition of the recording time of our spike data into equal intervals (i.e, time bins), on which the bin-based model (the almost exclusively applied model of synchrony in the neurobiology literature) relies in order to identify spike coincidences. The second one we consider, more in keeping with a time-continuous account of spiking activity, is a collection of sliding windows (one for each spike time) able to account for all spike coincidences in our spike trains that fall within the window length and that is consistent with the common, intended characterization of spike synchrony in the field, which regards two or more spikes as synchronous if they lie within a certain distance from each other (to be determined by the modeller). Second, in Section 3.2, we offer a* graded,* continuous alternative to some of the previous tests. In Section 4 we briefly discuss the complexity of computing the test statistics presented in the two previous sections. In Section 5 we evaluate the performance of our new test statistics on artificially generated collections of spike trains based on parameters learned from typical real recordings, compared to the performance of those in [20], and show that the former clearly outperform the latter. Finally, in Section 6 we summarize results.

## 2. Preliminary Definitions, Remarks, and Notation

Let *N* be our set of items (i.e., in our context, neurons). We will be working with parallel spike trains, one for each neuron in *N*, formalized as spike-time sequences (i.e., point processes) of the form {*t*_1_^*i*^,…, *t*_*k*_*i*__^*i*^}⊂(0, *T*], for *i* ∈ *N* and *T* ∈ *ℛ* (the recording time), where *k*_*i*_ is the number of times neuron *i* fires in the interval (0, *T*]. We denote the set of all these sequences by *𝒮*. Sets of sequences like *𝒮* constitute our raw data.

In order to identify (potential) assembly neurons and, ultimately, neuronal assemblies we need to determine first what constitutes spike synchrony: exact spike coincidences cannot be expected and thus an alternative, nontrivial characterization of synchrony is needed. Generally it is considered that two or more spikes are synchronous (or coincident)—that is, they constitute a synchronous event—if they lie within a certain (user-defined) distance from each other, say *w* ∈ *ℛ* ^+^. We will assume this notion of spike synchrony throughout.

**Figure 1:**
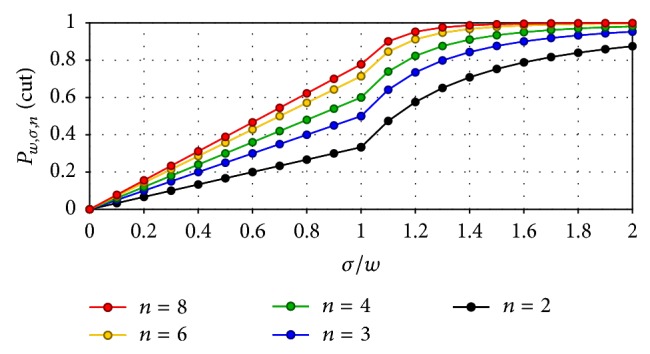
Probability that a group of *n* spikes (i.e., an *n*-spike coincidence) is cut by a bin boundary. The parameter *σ* is the scatter in the group (i.e., the time span or maximum distance that can exist between any two spikes in the group) and *w* is the bin width (i.e., time span within which we characterize synchrony). Probabilities are on the vertical axis.

The bin-based method, the almost exclusively applied method for dealing with synchronous spiking in the neurobiology literature, builds on the notion of synchrony above: the recording time is partitioned into time bins (i.e., windows) of equal length (*w* above, the time distance within which the modeller intends to define synchrony) and all those spikes that lie in the same time bin are regarded as synchronous. Notice though that the bin-based method can fail to identify some synchronous events: two or more spikes can be separated by a time distance way smaller than *w* and lie in two distinct time bins—what we called in other companion papers the* boundary problem*, which we addressed by means of an alternative method to identify and count spike coincidences which builds on an alternative window set, defined in the next section (that matches the intended characterization of spike synchrony given above), introduced in [17]. In order to illustrate the relevance of the boundary problem and the huge impact that time-bin boundaries have on the identification of synchrony we show, in Figure 1, the probability that spike coincidences of different sizes (with respect to different ratios between the scatter of the spikes—the time span of the spikes in the coincidence—and bin width) are cut by a time-bin boundary.

## 3. Statistics

In order to identify assembly neurons from *𝒮*-like data we propose here several statistics based on a variety of ideas (already briefly sketched in Section 1).

### 3.1. First Set: Window-Based Tests

We first present four test statistics that are based on counts of spike coincidences in a collection of (sliding) bins or windows *𝒳*. We denote the number of such windows by *W* (i.e., |*𝒳* | = *W*).

We denote by *W*_*I*_ the number of windows, where all neurons in a set *I*⊆*N* fire. To simplify we sometimes avoid set notation: instead of writing, for example, *W*_{*i*,*j*}_, we write *W*_*ij*_, for {*i*, *j*}⊆*N*.

*I*_*n*_⊆*N* is the subset of neurons that fire in the *n*th window.

*Conditional Pattern Cardinalities (CPC*_*1*_). This test (first introduced in [20] for time binning) builds on the idea that neurons participating in assemblies should have more neurons firing synchronously with them (due to the spikes of the other assembly neurons and the background spikes that are merely synchronous by chance) than it would be expected by chance under the assumption that they are not assembly neurons. Therefore, if *i* ∈ *N* belongs to a neuronal assembly, the average cardinality of the spike coincidences in which neuron *i* participates should be bigger than that expected by chance (i.e., under the assumption of independence).

In order to formalize our test statistic (*𝒯*_*α*_^cpc_1_^) we first define the amounts ${\bar{\mu }}_{\alpha }$ and *μ*_*α*_ as follows, for *i* ∈ *N*:

(1) μ¯αi=1W∑n=1WIn∖iα,μα(i)=1Wi∑n=1W1In(i)In∖iα,

where 1_*I*_*n*__ is the* indicator function* of the set *I*_*n*_ (i.e., 1_*I*_*n*__(*i*) = 1 if *i* ∈ *I*_*n*_ and 1_*I*_*n*__(*i*) = 0 otherwise) and *α* ∈ [1, *∞*) is a user-specified variable that, for values greater than 1, weights large cardinalities more strongly than smaller ones (on the understanding that mainly large cardinalities tell us about assembly activity while small ones can simply respond to chance events). In other words, ${\bar{\mu }}_{\alpha }$ is the unconditional average (for *α* = 1) pattern cardinality (taking all windows into account) while *μ*_*α*_ is the conditional average pattern cardinality given neuron *i* (i.e., conditional on neuron *i*: only windows containing a spike of neuron *i* are taken into account). If neuron *i* does not participate in an assembly the two averages should not differ significantly. However, if neuron *i* participates in an assembly then we would expect *μ*_*α*_ to be (significantly) larger. Therefore, by comparing the two averages we obtain a test for assembly participation. We formalize this comparison by defining the test statistic *𝒯*_*α*_^cpc_1_^ with respect to neuron *i* ∈ *N*, as follows:

(2) $$\begin{array}{c} T_{\alpha }^{\text{cp}{\text{c}}_{1}}\left(i\right)=\frac{\mu_{\alpha }\left(i\right)-{\bar{\mu }}_{\alpha }\left(i\right)}{{\bar{\mu }}_{\alpha }\left(i\right)}. \end{array}$$

*Conditional Item Frequencies (CIF*_*1*_). This test (first introduced in [20] for time binning) is based on the idea that, if *i* ∈ *N* belongs to one or more neuronal assemblies, it should fire more often with other neurons, namely, those that are also part of the assembly or assemblies, than it would be expected by chance under the assumption that it is not an assembly neuron.

For each neuron *j* (for *j* ≠ *i*) we consider *W*_*ij*_ the number of windows, where neurons *i*, *j* fire together and its expected number ${\hat{W}}_{ij}$ to build our test statistic (the latter is estimated as $W_{j}{\hat{\eta }}_{i}$, with ${\hat{\eta }}_{i}=W_{i}/W$—the estimated firing frequency of neuron *i*). If *W*_*ij*_ exceeds ${\hat{W}}_{ij}$ (significantly) then neurons *i*, *j* are likely to be part of the same assembly, due to which we see more cooccurrences of spikes of these two neurons that can be expected by chance. If, on the contrary, *W*_*ij*_ is less than ${\hat{W}}_{ij}$, it is highly likely that the observed cooccurrences are merely chance events. We formally express this intuition by means of our test statistic *𝒯*_*α*_^cif_1_^ as follows, for neuron *i*:

(3) $$\begin{array}{c} T_{\alpha }^{\text{ci}{\text{f}}_{1}}\left(i\right)=\frac{1}{|N|-1}\underset{j\in N\setminus \{i\}}{\sum }\zeta \left(W_{ij}>{\hat{W}}_{ij}\right){\left(W_{ij}-{\hat{W}}_{ij}\right)}^{\alpha }, \end{array}$$

where *ζ* is, here and throughout the rest of this section, a boolean operator that returns value 1 if the condition holds (i.e., in this statistic, if $W_{ij}>{\hat{W}}_{ij}$) and 0 otherwise (note that we are only interested in the former case, which could be indicative that neuron *i* belongs to an assembly). The value *α* ∈ [1, *∞*) offers the possibility of weighting large numbers of spike coincidences for pairs of the form {*i*, *j*} (over the expected ones) more than smaller ones.

*Conditional Item Weight (CIW*_*1*_). The previous test statistic (i.e., *𝒯*_*α*_^cif_1_^) was built based on the number of observed and expected spike coincidences of sets of the form {*i*, *j*} (where *i* is the neuron tested and *j* ∈ *N*∖{*i*}) without taking into account the cardinality of the sets *I*_*n*_ ⊂ *N* in the windows, where such {*i*, *j*}-coincidences occurred. It is plausible that {*i*, *j*}-coincidences that cooccur with many more spikes are more indicative of correlation (assembly activity) than only a few cooccurrences. Basically, in order to build this new statistical test, we combine the idea on which *𝒯*_*α*_^cpc_1_^ is based (i.e., that larger pattern cardinalities are possibly indicative of assembly activity) and that of *𝒯*_*α*_^cif_1_^ (i.e., that a neuron participating in an assembly fires more often together with some other specific neurons—those also in the assembly) and combine them by weighting spike cooccurrences with the corresponding pattern cardinality. This test statistic goes beyond what was presented in [20] and, given that we are bringing together two pieces of information that proved effective for our purposes (pattern cardinality and coincident spiking with other specific neurons), it can be expected to yield considerably better performance.

We formalize this idea by means of our test statistic *𝒯*_*α*_^ciw_1_^. In order to define such statistic we first need the values ${\bar{\omega }}_{ij}$ and *ω*_*ij*_ defined as follows:

(4) ω¯ij=∑n=1W1InjIn∖i,ωij=∑n=1W1Ini1InjIn∖i.

In other words, ${\bar{\omega }}_{ij}$ gives us the sum of the cardinalities of all sets of neurons in *I*_*n*_∖{*i*} that fire together with neuron *j* over our collection of windows *𝒳* (i.e., the occurrences of spikes of neuron *j* are weighted with the cardinality of the pattern in the window they appear in. Thus, ${\bar{\omega }}_{ij}$ is the total size of patterns containing a spike of neuron *j*). Similarly, *ω*_*ij*_ gives us the sum of the cardinalities of all sets of neurons in *I*_*n*_∖{*i*} that fire together with neurons *i* and *j* over *𝒳* (i.e., the cooccurrences of spikes of neurons *i*, *j* are weighted with the cardinality of the pattern in the window in which they occur).

We define the test statistic *𝒯*_*α*_^ciw_1_^, with a user-specified power *α*, as follows:

(5) $$\begin{array}{c} T_{\alpha }^{\text{ci}{\text{w}}_{1}}\left(i\right)=\frac{1}{\left|N\right|-1}\underset{j\in N\setminus \{i\}}{\sum }\zeta \left(\omega_{ij}>{\bar{\omega }}_{ij}{\hat{\eta }}_{i}\right){\left(\omega_{ij}-{\bar{\omega }}_{ij}{\hat{\eta }}_{i}\right)}^{\alpha }, \end{array}$$

with ${\hat{\eta }}_{i}=W_{i}/W$ the estimated firing frequency of neuron *i*. The parameter *α* ∈ [1, *∞*), as in previous statistics and in those that follow, offers the possibility of weighting larger (average) spike coincidences more than smaller ones.

*Conditional Pattern Overlap (CPO*_*1*_). While all preceding statistics were computed from aggregates over values computed from individual windows, for the test statistic we present now, we consider* pairs* of windows in which the neuron *i* ∈ *N* tested fires together with another set of neurons. The idea underlying this statistic is that cooccurrences of spikes of neuron *i* with those of any other neuron *j* (as considered in the two preceding statistics) may still be chance events. However, if spikes of several other neurons all occur together twice (as we look at pairs of windows) with spikes of the tested neuron *i*, this is a much stronger indicator of assembly activity. Apart from this difference, this statistic employs the same idea as *𝒯*_*α*_^ciw_1_^, only that the overlap of pairs takes the role of a single pattern.

We formalize this idea by means of the test statistic *𝒯*_*α*_^cpo_1_^, which we define as follows:

(6) Tαcpo1i=∑n=2 W∑m=1n−11In∩ImiζIn∩Im∖i>1    ·In∩Im∖iα,

where *ζ*(|*I*_*n*_∩*I*_*m*_∖{*i*}|>1) excludes patterns overlapping only in one neuron.

**Figure 2:**
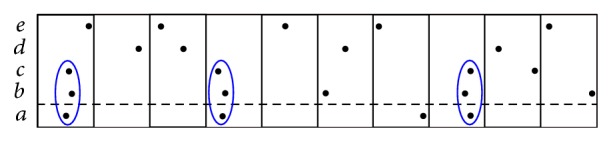
*Example*: A collection of spike trains for neurons *a*, *b*, *c*, *d*, *e* that contain a neuronal assembly formed by {*a*, *b*, *c*}—three injected coincidences in the example, circled in blue. The window set *𝒳* ^*b*^ (i.e., time binning) is considered in our example (yielding a partition with ten windows). We are interested in testing whether neuron *a* is part of an assembly. CPC_1_: in order to compute ${\bar{\mu }}_{1}(a)$ we consider the number of spikes of neurons *b*, *c*, *d*, *e* in each window and sum over. We get, for our example, ${\bar{\mu }}_{1}(a)=1.8$. We proceed in a similar way to assess *μ*_1_(*a*) by only considering those windows in which neuron *a* fires, which yields *μ*_1_(*a*) = 2. We thus get that *𝒯*_1_^cpc_1_^(*a*) = 1/9 (concluding that *a* is an assembly neuron depends on the significance of the value 1/9—see Section 5.1). CIF_1_: we have that *W*_*ab*_ = 3 and ${\hat{W}}_{ab}=2$ and that *W*_*ab*_ = 3 and ${\hat{W}}_{ab}=1.6$ (for the other two pairs—i.e., *a*, *d* and *a*, *e*—its number of coincidences is lower than its expected one under independence). These numbers yield *𝒯*_1_^cif_1_^(*a*) = 0.6. CIW_1_: on one hand we have, for the cardinalities of the patterns, where neuron *a* does not necessarily occur, ${\bar{\omega }}_{ab}=11$, ${\bar{\omega }}_{ac}=9$, ${\bar{\omega }}_{ad}=7$, and ${\bar{\omega }}_{ae}=9$ and, on the other hand, *ω*_*ab*_ = 6, *ω*_*ac*_ = 6, *ω*_*ad*_ = 0, and *ω*_*ae*_ = 3. For such values and ${\hat{\eta }}_{a}=0.4$ we have that *𝒯*_1_^ciw_1_^(*a*) = 1. CPO_1_: for this test statistic we only consider the windows that contain a spike of neuron *a*. Of those, only three of them—that is, those containing an instance of the assembly {*a*, *b*, *c*}—yield pairwise intersections of cardinality bigger than 1. Each such intersection contributes with a cardinality of 2 to the total value of our statistic, yielding *𝒯*_1_^cpo_1_^(*a*) = 6.

A simple example on how these test statistics that we have just presented are computed is given in Figure 2.

In Section 5 we report results on the evaluation of these statistical tests for two window sets of particular interest, which we denote by *𝒳* ^*b*^ and *𝒳*_*𝒮*_^*w*^ (except *𝒯*_*α*_^*cpo*_1_^, which was only evaluated on *𝒳* ^*b*^). “*b*” stands for “*bin*” and “*w*” for “(*sliding*)* window.*” The subscript *𝒮* reflects the dependence of *𝒳*_*𝒮*_^*w*^ on the underlying collection of spike trains *𝒮*:

- *𝒳* ^*b*^ is a partition (of intervals of length *w*, the time span within which we define spike synchrony) of the recording time *T*;
- *𝒳*_*𝒮*_^*w*^ is the set given by all the intervals of the form [*t* ^*i*^, *t* ^*i*^ + *w*], for all *t* ^*i*^ ∈ {*t*_1_^*i*^,…, *t*_*k*_*i*__^*i*^} (in *𝒮*) and all *i* ∈ *N*. The real value *w* refers to the particular (user-defined) time span.

Our definition of *𝒳* ^*b*^ is motivated by the bin-based model of synchrony that, as mentioned earlier, partitions the recording time *T* into time bins of equal length and counts as synchronous those spikes that lie in the same bin (which constitutes the most popular method for the identification of synchronous spiking in the neurobiology literature and the reference for the statistical tests presented in [20]). However, as we explained earlier (and illustrated by means of Figure 1), such an account of synchronous spiking leads to missing potential synchronous groups: groups of spikes that lie within the time span that determines synchrony (say *w*, as above)—and thus should be identified as synchronous—but that, due to the placement of the bin boundaries, fall into different time bins and are thus not reported as synchronous by the bin-based model. In order to bring more flexibility to the placement of the bin boundaries and this way achieve a better account of spike synchrony some possibilities come naturally to our mind. Maybe the most natural way would be to look at each spike and check its neighborhood, considering a time span *w*/2 in each direction (i.e., considering the window [*t* − *w*/2, *t* + *w*/2], for *t* the corresponding spike time). However, this has the disadvantage that looking only at *w*/2 in each direction may still miss synchronous spiking, hence the natural possibility of considering a neighborhood with span *w* in each direction, but this increases the number of chance occurrences. The next option is then to let a window (of length *w*) slide over the spike trains stopping at each spike, which captures each spike coincidence in the range given by *w* at least once. Such a collection of windows is given by *𝒳*_*𝒮*_^*w*^.

### 3.2. Second Set: Time-Continuous Approach

In this section we offer a* continuous* version of some of the previous statistical tests that are implicitly built on a* graded*, continuous notion of spike synchrony.

We consider, for each spike *t* ^*i*^ ∈ {*t*_1_^*i*^,…, *t*_*k*_*i*__^*i*^} and *i* ∈ *N*, an* influence region* that corresponds to the distance within which two or more spikes are regarded as synchronous (i.e., for a time span *w* ∈ *ℛ* ^+^, we would define the influence region of spike *t* ^*i*^ as the interval [*t* ^*i*^ − *w*/2, *t* ^*i*^ + *w*/2]). From the influence region we define the function *f* ^*i*^ as follows:

(7) $$\begin{array}{c} f^{i}\left(x\right)=\left\{\begin{array}{cc} 1, & \text{if}\;\;x\in \left[t^{i}-\frac{w}{2},t^{i}+\frac{w}{2}\right],\\ 0, & \text{otherwise}. \end{array}\right. \end{array}$$

In what follows we will represent spikes by these maps (i.e., *t* ^*i*^ will be represented by *f* ^*i*^ above). We call functions of this form* influence maps* (and the windows of the form [*t* ^*i*^ − *w*/2, *t* ^*i*^ + *w*/2] underlying them are called* influence regions*). Such functions constitute the building blocks of the synchrony model that we introduce in our companion paper [21], which is characterized by a* graded* notion of synchrony (which differs substantially from the intended notion of synchrony in this paper, which is bivalent): the degree of synchrony among two or more spikes is defined as the integral (i.e., area) of the intersection of their corresponding influence maps. Such degree is thus a value in the interval [0,1] (e.g., 0 if the time distance between any two spikes is greater than or equal to *w* and 1 if there is exact time synchrony between them).

Next we define *ℱ* ^*i*^ as follows:

(8) $$\begin{array}{c} F^{i}\left(x\right)=\underset{n\in \left\{1,\ldots ,k_{i}\right\}}{\max}f_{n}^{i}\left(x\right), \end{array}$$

where *f*_*n*_^*i*^ is the map corresponding to spike *t*_*n*_^*i*^. In other words, any spike time that lies in an interval of the form [*t* ^*i*^ − *w*/2, *t* ^*i*^ + *w*/2], for *t* ^*i*^ a spike of neuron *i* ∈ *N* (and that, thus, should be regarded as synchronous with *t* ^*i*^), will be given, by *ℱ* ^*i*^(*x*), value 1.

#### 3.2.1. Conditional Pattern Cardinalities (CPC_2_)

We introduce now a continuous version of the test statistic *𝒯*_*α*_^cpc_1_^, in terms of influence regions and influence maps, which we denote by *𝒯*_*α*_^cpc_2_^.

Formally, for *i* ∈ *N*, we define the values ${\bar{\mu }}_{\alpha }$ and *μ*_*α*_ as follows:

(9) μ¯αi=1T∫0,T∑j∈N∖iFjxαdx,μα(i)=1s(Ri)∫Ri∑j∈N∖iFjxαdx,

with

(10) $$\begin{array}{c} s\left(R_{i}\right)=\int_{\left(0,T\right]}F^{i}\left(x\right)dx. \end{array}$$

Here *α* ∈ [1, *∞*) is, as in previous statistics and all others that we will be presenting in this section, a weighting parameter that, for values greater than 1, weights large spike coincidences more strongly than smaller ones. As with *𝒯*_*α*_^cpc_1_^, ${\bar{\mu }}_{\alpha }$ and *μ*_*α*_ measure* average* spike cardinalities (notice that

(11) $$\begin{array}{c} \underset{j\in N\setminus \{i\}}{\sum }F^{j}\left(x\right) \end{array}$$

gives us, at each *x*, the number of influence regions corresponding to spikes of neurons in *N*∖{*i*} that overlap and, thus, the number of spikes that lie in the window [*x* − *w*/2, *x* + *w*/2]). As with ${\bar{\mu }}_{\alpha }$ and *μ*_*α*_ in *𝒯* ^cpc_1_^, we expect *μ*_*α*_(*i*) to be bigger than ${\bar{\mu }}_{\alpha }(i)$ if *i* ∈ *N* is an assembly neuron. Based on this intuition, we formally define the test statistic *𝒯*_*α*_^cpc_2_^ as follows, for *i* ∈ *N*:

(12) $$\begin{array}{c} T_{\alpha }^{\text{cp}{\text{c}}_{2}}\left(i\right)=\frac{\mu_{\alpha }\left(i\right)-{\bar{\mu }}_{\alpha }\left(i\right)}{{\bar{\mu }}_{\alpha }\left(i\right)}. \end{array}$$

#### 3.2.2. Conditional Item Frequencies (CIF_2_)

We present now an adaptation of the test statistic *𝒯*_*α*_^cif_1_^ to influence maps and a continuous domain, which we will denote by *𝒯*_*α*_^cif_2_^, and that responds to the same ideas as *𝒯*_*α*_^cif_1_^.

For each neuron *j* we define the values *L*_*ij*_ and ${\hat{L}}_{ij}$ as follows:

(13) Lij=∫0,TFixFjxdx,L^ij=1T∫0,TFixdx∫0,TFjxdx.

We formally define the statistic *𝒯*_*α*_^cif_2_^ as follows, for neuron *i*:

(14) $$\begin{array}{c} T_{\alpha }^{\text{ci}{\text{f}}_{2}}\left(i\right)=\frac{1}{\left|N\right|-1}\underset{j\in N\setminus \left\{i\right\}}{\sum }\zeta \left(L_{ij}>{\hat{L}}_{ij}\right){\left(L_{ij}-{\hat{L}}_{ij}\right)}^{\alpha }, \end{array}$$

where *ζ* is the boolean operator returns value 1 if $L_{ij}>{\hat{L}}_{ij}$ and 0 otherwise.

#### 3.2.3. Conditional Item Weight (CIW_2_)

A continuous version of the test statistic *𝒯*_*α*_^ciw_1_^ is that which we denote by *𝒯*_*α*_^ciw_2_^.

In order to formalize our continuous version of the statistic we first define the values ${\bar{\omega }}_{ij}$ and *ω*_*ij*_ as follows:

(15) ω¯ij=∫0,TFjx∑k∈N∖iFkxdx,ωij=∫0,TFixFj(x)∑k∈N∖{i}Fk(x)dx.

We define *𝒯*_*α*_^ciw_2_^ as follows, for neuron *i*:

(16) $$\begin{array}{c} T_{\alpha }^{\text{ci}{\text{w}}_{2}}\left(i\right)=\frac{1}{\left|N\right|-1}\underset{j\in N\setminus \left\{i\right\}}{\sum }\zeta \left(\omega_{ij}>{\bar{\omega }}_{ij}{\hat{\eta }}_{i}\right){\left(\omega_{ij}-{\bar{\omega }}_{ij}{\hat{\eta }}_{i}\right)}^{\alpha }, \end{array}$$

where ${\hat{\eta }}_{i}$ is the frequency *s*(*R*_*i*_)/*T*.

As before, *ζ* is the boolean operator returns value 1 if $\omega_{ij}>{\bar{\omega }}_{ij}$ and 0 otherwise.

## 4. Computational Complexity

In this section we briefly analyze the complexity of computing our statistics.

First of all, if we take as reference the window set *𝒳* ^*b*^ (i.e., binning), we have that CPC_1_, CIF_1_, and CIW_1_ are linear in the number of windows in *𝒳* ^*b*^. Also, as it is probably clear, CPC_1_ is constant in the number of neurons (only the pattern cardinality is taken into account; the composition of the pattern itself is irrelevant) and CIF_1_ and CIW_1_ are linear (since one needs to loop over the neurons). More formally, we have that the complexity of computing CPC_1_ is at most of the order *O*(*k*), where *k* is the number of time bins, and that the complexity of CIF_1_ and CIW_1_ is of the order *O*(*s* + *n*), where *s* is the number of spikes and *n* is the number of neurons. As for CPO_1_, it is quadratic in the number of time bins and linear in the number of neurons. More formally, we have that its complexity is of the order *O*(*n* *k* ^2^) (this bound could be reduced by the size of the largest set of neurons that fires together in a window, which would replace *n*). If, instead, we consider the window set *𝒳*_*𝒮*_^*w*^ then we have that the computation of CPC_1_, CIF_1_, and CIW_1_ is linear in the total number of spikes and that CIF_1_ and CIW_1_ are also linear in the number of neurons. Formally, the complexity of CPC_1_ is of the order *O*(*s*) and that of CIF_1_ and CIW_1_ is of the order *O*(*n* *s*) (where, as before, *n* could be replaced by the largest number of neurons firing together in a window). The statistics CPC_2_, CIF_2_, and CIW_2_ have the same complexities as its window-based counterparts.

## 5. Evaluation

In this section we show some results concerning the evaluation of our statistical tests on artificially generated collections of spike trains. Such artificially generated collections, in which all assemblies—and thus assembly neurons—are known, are necessary in order to assess whether our test statistics do what they are supposed to do which is to identify all assembly neurons and discard all those that are not. Only on such data a proper evaluation of our test statistics is possible.

For the results reported in this section we generate our collections of spike trains as follows: for each signature

(17) $$\begin{array}{c} \langle z,c\rangle \in \left\{3,\ldots ,12\right\}\times \left\{3,\ldots ,12\right\}, \end{array}$$

(where *z* stands for the size of the neuronal group and *c* for the number of spike coincidences injected) we generate 1000 trials, each consisting of 100 spike trains (one for each neuron) independently generated as 3-second Poisson processes (i.e., *T* = 3) of constant rate 20 Hz (which represent the background activity), with *c* injected spike coincidences of a particular *z*-neuron pattern containing the neuron we are testing for (for the neurons with injected synchronous spikes, a corresponding number of background spikes were removed and thus the background firing of the assembly neurons was adjusted accordingly). In order to generate such coincidences a random choice of *c* points in the interval (0, *T*] is considered for each trial and added to the background spiking activity. In trials with nonexact coincidences (i.e.,* jittered* trials, as opposed to* nonjittered* trials with exact coincidences) a random shift is added, which we model by means of a uniform random variable on the interval [−0.0015,0.0015] (i.e., ±1.5 maximal millisecond shift, in keeping with the time span *w* = 0.003 and the corresponding length of windows and influence regions that we are considering for our statistics). More results and diagrams corresponding to artificially generated data with slightly different settings can be found in http://www.borgelt.net/docs/napa.pdf. The general conclusions that could be drawn from them do not differ from those reported here.

### 5.1. Significance

To estimate the distribution of the test statistics we generate surrogate data from our original spike trains as follows: modifications of the original data that are intended to keep all its essential features except synchrony among the neuron we are testing and the others (see, e.g., [22] or [23] for a survey and analysis of methods to generate surrogate data from parallel spike trains). In order to keep as many properties of the original data as possible we create only a surrogate train for the neuron we are currently testing, which replaces the original train. The trains of all other neurons are left unchanged. With the surrogate train the test statistic is recomputed. Generating a surrogate train and recomputing the test statistic are repeated 1000 times, in order to obtain an estimate of the distribution of the test statistic. We then determine the fraction of surrogate trains that produced a test statistic value exceeding the one obtained with the actual (real) train and thus obtain a *P* value. Note that, for testing another neuron, the original (real) train of any neuron tested before is used. That is, no surrogate trains are evaluated for neurons other than the one to be tested.

### 5.2. Results

**Figure 3:**
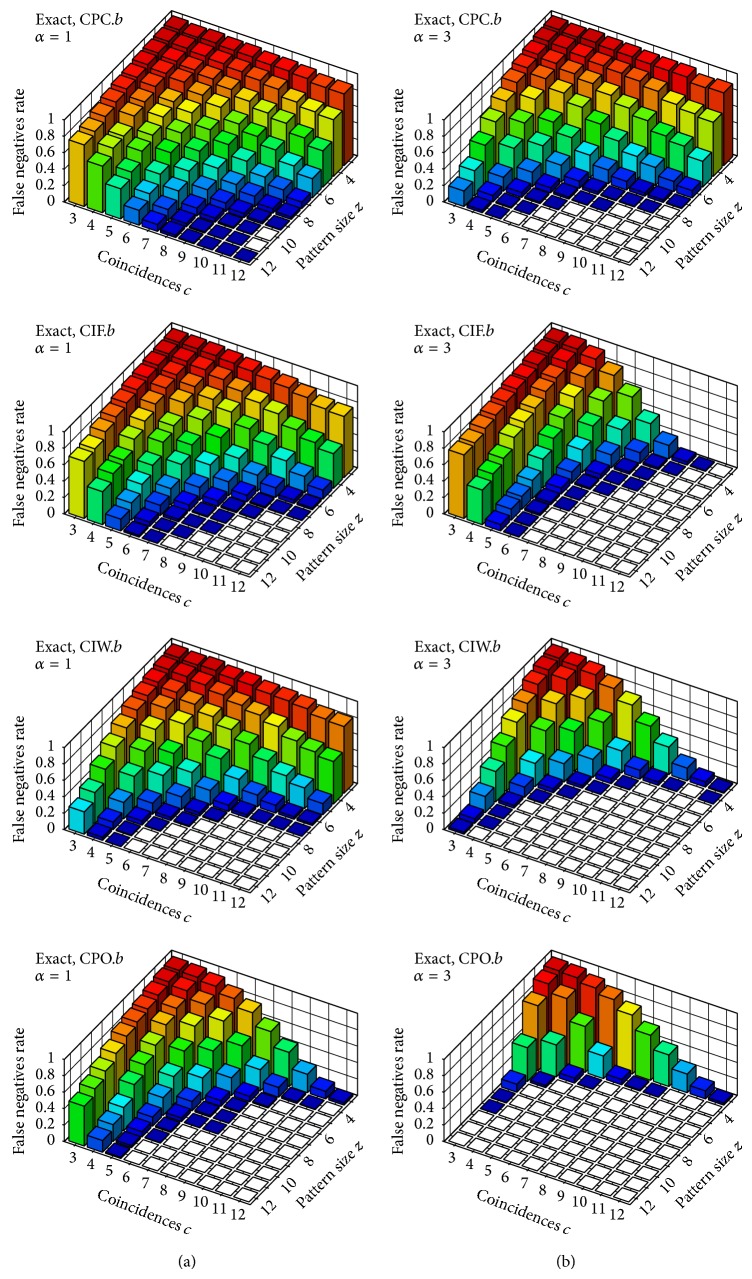
Rate of* false negatives* on nonjittered trials (i.e., with exact coincidences). Test statistics CPC_1_, CIF_1_, CIW_1_, and CPO_1_ with respect to the window set *𝒳* ^*b*^ (i.e., binning). Column (a) shows results for the parameter *α* = 1 and column (b) for *α* = 3.

**Figure 4:**
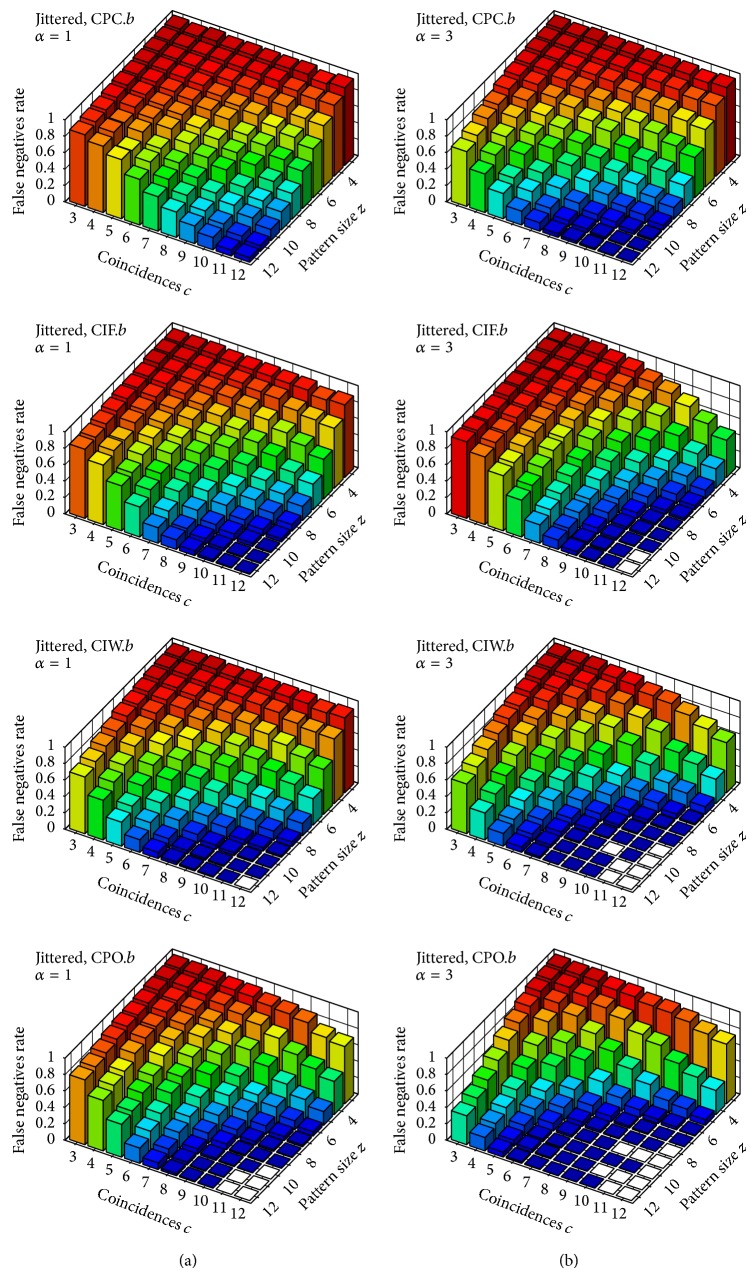
Rate of* false negatives* on jittered trials (i.e., with nonexact coincidences). Test statistics CPC_1_, CIF_1_, CIW_1_, and CPO_1_ with respect to the window set *𝒳* ^*b*^ (i.e., binning). Column (a) shows results for the parameter *α* = 1 and column (b) for *α* = 3.

**Figure 5:**
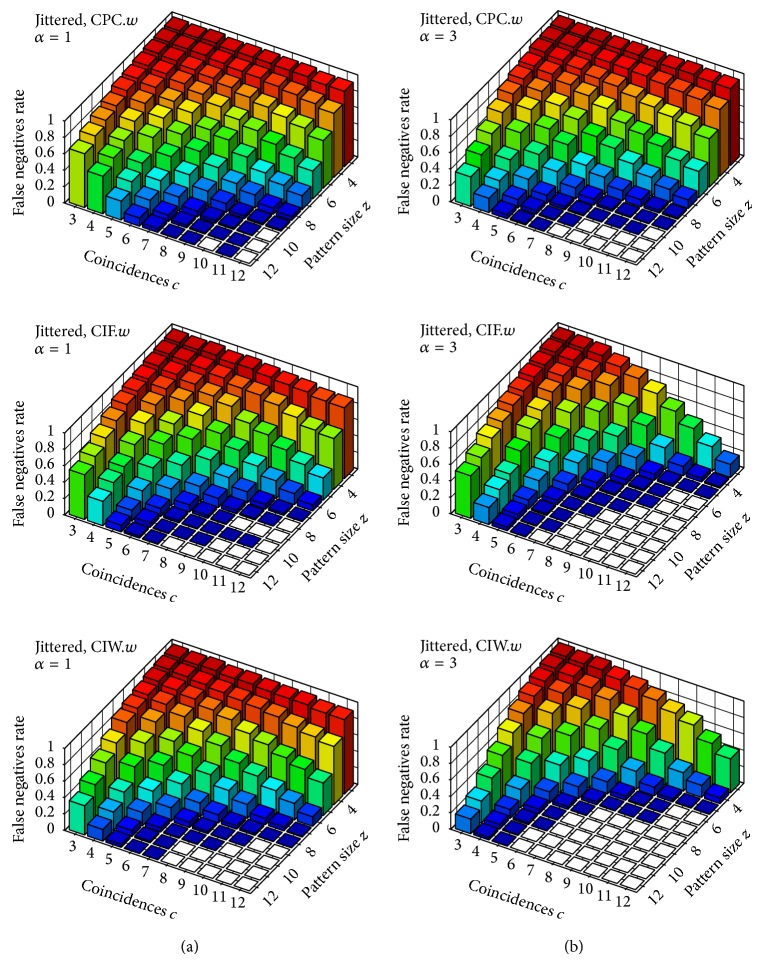
Rate of* false negatives* on jittered trials (i.e., with nonexact coincidences). Test statistics CPC_1_, CIF_1_, and CIW_1_ with respect to the window set *𝒳*_*𝒮*_^*w*^ (i.e., sliding window). Column (a) shows results for the parameter *α* = 1 and column (b) for *α* = 3.

**Figure 6:**
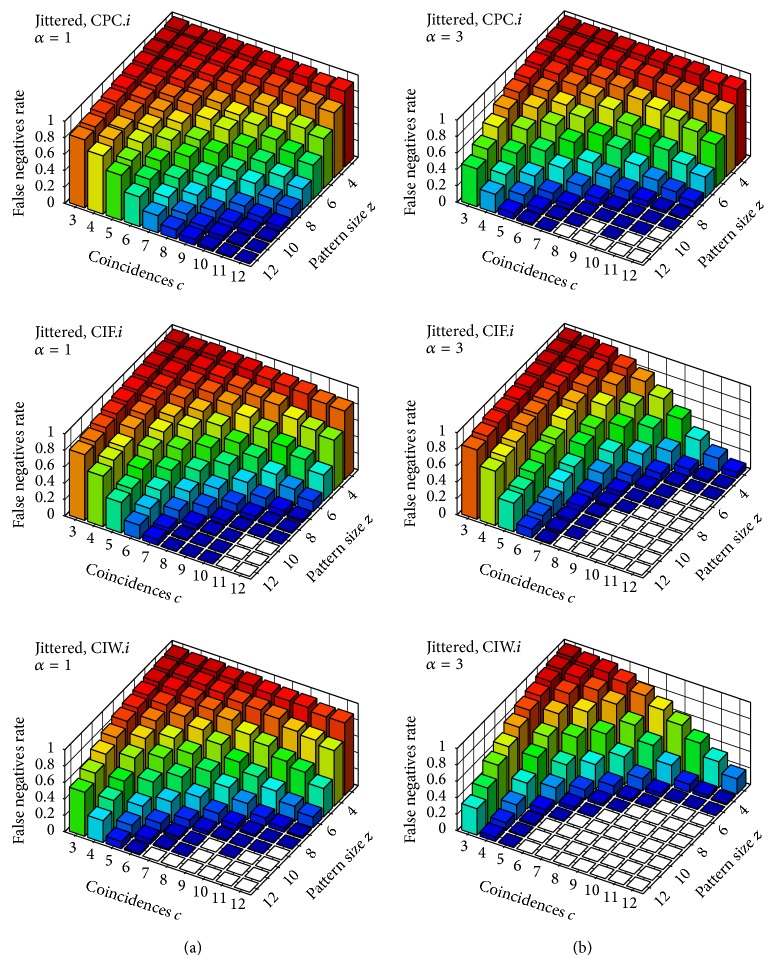
Rate of* false negatives* on jittered trials (i.e., with nonexact coincidences). Test statistics CPC_2_, CIF_2_, and CIW_2_. Column (a) shows results for the parameter *α* = 1 and column (b) for *α* = 3.

Figures 3–6 feature diagrams with rates of* false negatives* for each signature 〈*z*, *c*〉, with *z*, *c* ∈ {1,…, 12} over the 1000 trials; that is, the rate of trials (over 1000) in which the tested neuron that belongs to the group with injected coincidences is not identified as an assembly neuron—on the understanding that a group of neurons of size at least 3 with at least 3 spike coincidences in our trials constitutes a potential neuronal assembly (see, e.g., [17] or [18] for a better insight). Maybe it is worth stressing that, if we were to test a neuron that does not belong to an assembly, it would be identified by our test statistics as an assembly neuron (i.e., a* false positive*) in about 1% of our trials (which is probably clear, since this is our significance level, learned from uncorrelated trials).

In Figure 3 we show results for the window-based statistics CPC_1_, CIF_1_, CIW_1_, and CPO_1_ on *𝒳* ^*b*^ (i.e., when considering time binning for the identification of spike coincidences). The first two test statistics (i.e., CPC_1_ and CIF_1_) were already introduced and evaluated in a companion paper ([20]) on artificially generated trials based on slightly different—but essentially comparable—settings. As the diagrams in Figure 3 show, the two new test statistics CIW_1_ and CPO_1_ introduced in this paper report considerably lower rates of false negatives than those already introduced in [20] on nonjittered trials (the best performance being that of CPO_1_ that, as was seen in the previous section, is more costly than the other three in terms of computational efficiency). The performance of all such statistics with respect to *α* = 3 tends to be substantially better than statistics with *α* = 1 for most signatures: for CPC_1_ an increase in the exponent *α* yields an increase in sensitivity towards smaller patterns (i.e., towards smaller values for *z*) while for CIF_1_ such an increase yields an improvement in sensitivity towards a smaller number of coincidences (i.e., towards smaller values for *c*). CIW_1_ combines both effects, since it combines pattern cardinality assessment and coincidence counts (which is precisely what was intended with the definition of this statistic). The effect of *α* on CPO_1_ is even higher, since it exploits cooccurrences not only of pairs but of larger groups of neurons.

Figure 4 shows results for the same window-based statistics on jittered trials. As can be expected, the performance of all such statistics worsens substantially when dealing with nonexact spike coincidences. This is due to the above mentioned boundary problem when using the window set *𝒳* ^*b*^ (i.e., binning): two or more spikes can be less than *w* milliseconds apart (in our evaluations *w* = 0.003) but still lie in different windows and thus be regarded as nonsynchronous (a detailed analysis and* quantification* of the effect of the boundary problem can be found in our companion paper [24]).

In order to improve performance when dealing with nonexact spike coincidences and to overcome the boundary problem in binning we introduced an alternative window set *𝒳*_*𝒮*_^*w*^ for our window-based statistics and a time-continuous alternative to them by means of our test statistics CPC_2_, CIF_2_, and CIW_2_. Diagrams in Figure 5 show the performance of our window-based statistics CPC_1_, CIF_1_, and CIW_1_ on *𝒳*_*𝒮*_^*w*^ (CPO_1_ becomes very inefficient on *𝒳*_*𝒮*_^*w*^—due to the much larger number of windows and its quadratic complexity in the number of windows—and thus was not tested). Performance of such statistics on *𝒳*_*𝒮*_^*w*^ is, for most signatures, better than the corresponding performance on *𝒳* ^*b*^ (more so with respect to *α* = 3). As was mentioned, such improvement is mostly due to the fact that, by considering *𝒳*_*𝒮*_^*w*^ in place of *𝒳* ^*b*^, we identify all injected coincidences.

Diagrams in Figure 6 show the performance of our test statistics CPC_2_, CIF_2_, and CIW_2_. Overall, the performance of our window-based statistics on *𝒳*_*𝒮*_^*w*^ and the corresponding time-continuous statistics (based on influence regions and influence maps) are not clearly distinguishable from the diagrams and, among all test statistics introduced in this paper, CIW_1_ and CIW_2_ seem to yield the best results.

We are currently exploring possibilities to transfer the ideas on which CPO_1_ is based to work with *𝒳*_*𝒮*_^*w*^ without incurring quadratic computational complexity and also in the time-continuous approach. Although it is unclear how the statistic could be expressed in terms of influence regions (in the time-continuous approach), with such a transfer one can hope to achieve even better performance, as was seen for CPO_1_ when working with *𝒳* ^*b*^.

## 6. Conclusion

In this paper we have presented several test statistics to identify assembly neurons from multiple-electrode recordings. The aim of such statistics is to reduce the set of neurons to a relevant subset of them and in this way ease the task of identifying neuronal assemblies in further analyses (a task which, due to the large amount of neurons that can nowadays be recorded, is undermined by the computational explosion that comes from having to consider every possible subset of them as a potential neuronal assembly).

We have provided two types of statistics as follows: the window-based statistics (CPC_1_, CIF_1_, CIW_1_, and CPO_1_) and the time-continuous statistics (CPC_2_, CIF_2_, and CIW_2_). The former rely on a window-based approach to identify spike coincidences and the latter on what we called influence regions (i.e., a time span around each spike within which synchrony with other spikes is defined—two or more spikes are synchronous in these settings if their influence regions overlap). For the window-based statistics we considered two window sets in our evaluations as follows: a partition of the recording time of our spike data into equal intervals (which is called* binning*)—on which the bin-based model of synchrony relies in order to identify spike coincidences—and a collection of sliding windows (one for each spike time), able to account for all spike coincidences in our spike trains that fall within the window length, which is more in keeping with the common, intended characterization of spike synchrony in the field, which regards two or more spikes as synchronous if they lie within a certain distance from each other.

Two of the window-based statistics (CPC_1_ and CIF_1_) were first presented and evaluated with binning in a companion paper ([20]) for artificially generated* nonjittered* trials (i.e., with exact spike coincidences injected). In this paper we have shown that the two novel window-based statistics here presented (i.e., CIW_1_ and CPO_1_) perform substantially better in such settings, in terms of rates of false negatives. Performance of the latter is still better on jittered trials, yet, in these settings, test statistics based on the sliding-window set and the time-continuous ones yield much better results, as was shown.
